# STDP, Hebbian cell assemblies, and temporal coding by spike synchronization

**DOI:** 10.1186/1471-2202-12-S1-P142

**Published:** 2011-07-18

**Authors:** Andreas Knoblauch, Florian Hauser

**Affiliations:** 1 Honda Research Institute EU, 63073 Offenbach/Main, Germany; 2 Institute of Neural Information Processing, Ulm University, 89069 Ulm, Germany

## 

The question whether neural activity follows a rate code or a temporal code is still an unsolved issue in neuroscience. Recent theoretical works have suggested that realistic models of spike-timing-dependent-plasticity (STDP) in recurrent networks could resolve this issue because they observed a seemingly unique relation between the neural code and the resulting pattern of synaptic connections [1]. In particular, these works argue that temporal coding would be inconsistent with bidirectional synaptic connectivity as observed in visual cortex [2] and expected for Hebbian cell assemblies (“what fires together wires together”). This conclusion is based on two assumptions, first, that simple STDP based on spike doublets would be generally unable to produce bidirectional synaptic connectivity (see references in [1]) and, second, that realistic voltage-based STDP would be able to produce bidirectional synaptic connectivity only for rate coding where signals are coded by cell ensembles jointly elevating firing rates on a time scale of hundreds of milliseconds [1].

We have disproved both assumptions. In particular, here we show that already simple STDP models can easily grow bidirectional connections for a temporal code based on spike synchronization unless synchronization is extremely precise (see [3] for disproving the second assumption). To this end we present simulation results for various STDP models [4] including all-to-all (AA) doublet STDP, nearest-neighbor (NN) doublet STDP, and various triplet STDP model variants with parameters fitted to experimental data and including realistic propagation delays. It turns out that all model variants can grow functional cell assemblies as reflected by strong bidirectional connectivity and tested within an associative memory framework [5]. This requires coarsely synchronized inputs such that spikes of the neurons within a cell assembly get synchronized on a time scale of 5 to 50 milliseconds, whereas more precise synchronization leads to depression of synaptic weights and, thus, destruction of the cell assembly. For NN doublet STDP and triplet STDP synaptic potentiation is boosted by increasing the rate of synchronized inputs, whereas high rates destroy cell assemblies for AA doublet STDP.

Thus, both rate coding and temporal coding based on coarse synaptic synchronization can account for the bidirectional connectivity observed, for example, in visual cortex [2]. However, we argue that the temporal code will be much more energy efficient for learning because it allows to grow and preserve cell assemblies at low mean firing rates at the level of spontaneous activity, whereas a rate code (based on uncorrelated Poissonian firing) can grow cell assemblies only by maintaining high firing rates over longer time intervals. Moreover, additional simulation experiments demonstrate how STDP can quickly grow multiple independent cell assemblies reflecting the spike timing relations of simultaneously active input neurons. We speculate that such a multiplexing mechanism may play a role both for working memory and long-range communication where “copies” or “indices” [6] of cell assemblies could easily be transmitted between cortical areas.
